# Supplementary material for: Strain-driven domain wall network with chiral junctions in an antiferromagnet
Source: Nat Commun. 2025 Nov 29;16:10808. doi: 10.1038/s41467-025-66700-0 (PMC12669766; doi:10.1038/s41467-025-66700-0)
Supplement: Supplementary file 1 — Supplementary Information [file 41467_2025_66700_MOESM1_ESM.pdf]

Supplementary Information for: Strain-driven domain wall network with chiral junctions in an antiferromagnet

Vishesh Saxena,[1] Mara Gutzeit,[2] Arturo Rodríguez-Sota,[1] Soumyajyoti Haldar,[2] Felix Zahner,[1] Roland Wiesendanger,[1] André Kubetzka,[1] Stefan Heinze,[2,3] and Kirsten von Bergmann[1]

[1]*Institute of Nanostructure and Solid State Physics, University of Hamburg, Jungiusstrasse 11, 20355 Hamburg, Germany*
[2]*Institute of Theoretical Physics and Astrophysics, University of Kiel, Leibnizstrasse 15, 24098 Kiel, Germany*
[3]*Kiel Nano, Surface, and Interface Science (KiNSIS), University of Kiel, Germany*

SUPPLEMENTARY NOTE 1: COMPUTATIONAL DETAILS

For structural relaxations, we have used symmetric films comprising nine Ir layers and Mn double-layers (DL) on both sides of the film. The initial interlayer distance, 2.25 Å, was set using the equilibrium lattice parameter of the Ir bulk obtained in DFT within the generalized gradient approximation (GGA). Only the z coordinates of the Mn DL as well as the first two Ir layers were allowed to relax, while the five central Ir layers were kept fixed to the bulk value. The two-dimensional (2D) Brillouin zone (BZ) of both magnetic unit cells was sampled by a 15×15×1 $\overline{\Gamma}$-centered k-mesh. According to experimental observations [1], the subsurface Mn layer only grows in hcp stacking on the Ir(111) surface.

Since closely related systems as for instance a Mn monolayer on the Re(0001) surface [2] are known to exhibit a strong antiferromagnetic (AFM) behaviour, we have considered both ferromagnetic (FM) and row-wise antiferromagnetic (RW-AFM) states. The geometry optimization for the FM and the layered antiferromagnetic (LAFM) state, i.e. with the magnetic moments being oriented parallel within one layer and antiparallel between both layers, was carried out within a one-atom 2D unit cell per layer, whereas for both possibilities of the RW-AFM state a 2D magnetic unit cell with two atoms (Fig. S3) was applied. The total energies with respect to the RW-AFM$_{\rightleftarrows}$ state are given in Table S1. The magnetic moments of the four considered collinear spin configurations for the two Mn DLs on Ir(111) after the optimization process are listed in Table S2. With regard to the relaxed interlayer distances, only the respective values for the RW-AFM$_{\rightleftarrows}$ state are given in Table S3 since this spin structure has turned out to be energetically lowest among the four possibilities.

Consequently, its parameters serve as a basis for calculating the total energies of the two possible non-collinear 3Q states of the hcp-Mn/hcp-Mn/Ir(111) film system. Note that both in the main text as well as in this supplementary information we mainly focus on the results obtained for both Mn layers in hcp stacking; however, as mentioned below we obtain qualitatively similar results for fcc-Mn/hcp-Mn/Ir(111). Here, asymmetric films consisting of nine Ir layers and one Mn DL were used and a 15×15×1 Monkhorst-Pack (MP) k-mesh was applied to sample the 2D BZ of the 4-atomic hexagonal surface unit cell indicated in Fig. S3. These noncollinear DFT calculations were performed self-consistently via the constrained local moment approach, i.e. the directions of the magnetic moments within the unit cell were kept fixed, while their magnitudes were allowed to relax.

We have also considered a full relaxation of the two Mn and the first two Ir layers for the RW-AFM$_{\rightleftarrows}$ state of hcp-Mn/hcp-Mn/Ir(111). Our calculations indicate a lateral shift of the Mn rows of the surface layer along the $[11\overline{2}]$ direction. The relaxed interlayer distances as well as the amount of lateral shift of the Mn atoms are given in Table S3. In addition, we performed a similar calculation for the FM, LAFM and RW-AFM$_{\rightrightarrows}$ state, i.e. for a net FM interlayer coupling of the magnetic moments, of hcp-Mn/hcp-Mn/Ir(111) in order to check whether the effect of the lateral shift is restricted to the RW-AFM$_{\rightleftarrows}$ state. These results are summarized in Table S4 indicating that significant lateral changes indeed only occur for the RW-AFM$_{\rightleftarrows}$ state for which the magnetic moments couple antiferromagnetically between the two layers. Interestingly, the large lateral displacement of the RW-AFM$_{\rightleftarrows}$ state does not depend on the stacking of the upper Mn layer; as test calculations for the fcc-Mn/hcp-Mn/Ir(111) system indicate, the atoms of the fcc-stacked Mn layer adopt the same surface structure as those of the initially hcp-stacked configuration (see Fig. 3e of the main text) resulting in the same magnetic state and thereby confirming the exchange interaction between the Mn layers as the origin of the novel effect reported in this work.

The Mn DL on Ir(111) is characterized by a strong AFM interlayer Heisenberg exchange coupling mediated by nearest neighbors of the two magnetic layers which can already be concluded from the total energy differences of the four collinear states discussed so far. A determination of inter- and intralayer exchange constants can be obtained

from spin spiral calculations [3]. Therefore, the DFT calculated structural symmetry breaking of the upper Mn layer is induced by the exchange interaction. This explanation is supported by the above-mentioned test calculations for the collinear FM, LAFM and RW-AFM$_{\rightrightarrows}$ state of the system. While the former two do not show any influence of a lateral displacement, the RW-AFM$_{\rightrightarrows}$ structure with its net FM coupling of the moments between the two layers also experiences a lateral shift (Fig. 3f in the main text). However, the effect is much less pronounced than for the RW-AFM$_{\rightleftarrows}$ state with the Mn atoms of the top layer being displaced by only 0.26 Å and a much smaller energy gain of about 17 meV/Mn atom with respect to the unshifted state (Fig. 3d in the main text). As seen in Fig. 3f of the main text, in the RW-AFM$_{\rightrightarrows}$ state only the antiferromagnetic interaction of a Mn top layer atom with one Mn neighbor from the bottom layer can be strengthened by the lateral shift. This can explain the smaller shift and energy gain. Note that for the noncollinear 3Q state a lateral shift is prohibited by symmetry; for the sake of completeness, we have verified this presumption from a respective DFT calculation.

To demonstrate the independence of the energy gain obtained for the lateral shifted RW-AFM$_{\rightleftarrows}$ state of hcp-Mn/hcp-Mn/Ir(111) from the interlayer distances calculated in different ways (see second and third line of Table S3) and to be consistent with the results on the 3Q state, we have computed the total energy of the shifted structure in the two-atomic unit cell using the relaxed geometry of the original RW-AFM$_{\rightleftarrows}$ state (second line of Table S3). Here, again an asymmetric slab with nine Ir substrate layers and a 15×15×1 $\overline{\Gamma}$-centered k-mesh was chosen. The energy difference with respect to the unshifted RW-AFM$_{\rightleftarrows}$ state obtained in this way amounts to −50 meV/Mn atom confirming the energy gain to be caused mainly by the lateral displacement of the Mn atoms in the top layer (cf. −57.36 meV/Mn atom given in Table S4). In order to apply strain in the RW-AFM state in the Mn DL on Ir(111) we have changed the unit cell lengths along the respective strain directions (see Fig. S7). The 2D unit cells used for these DFT calculations are marked in Fig. S7b. For strain direction 1 and strain direction 2, we used a 16×16×1 and 7×12×1 k-mesh, respectively. All other computational parameters were unchanged, including the number of Ir substrate layers and the asymmetric film setup.

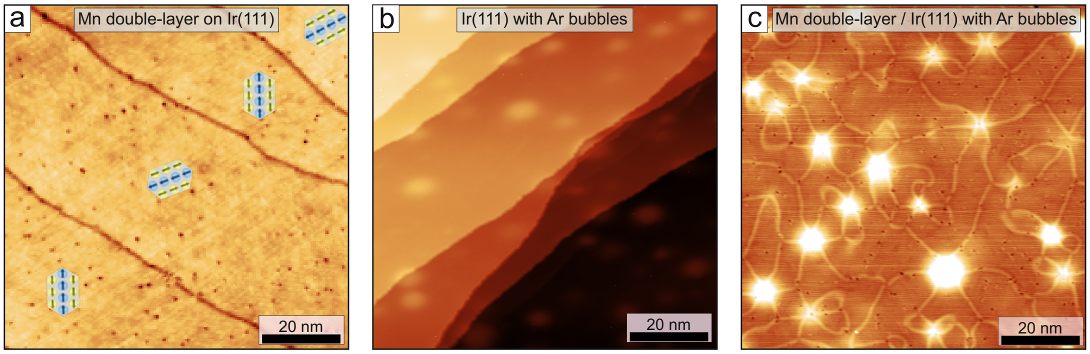

Figure S1. **Impact of Ar bubbles on domain wall density.** **a**, Constant-current STM image of the Mn double-layer on a broad Ir(111) terrace ($\Delta z = 25$ pm). In contrast to the other samples with Ar bubbles, in this preparation the Ir(111) substrate was annealed at a higher temperature of $T \approx 1600\,\mathrm{K}$ before the deposition of Mn. Dark lines indicate domain walls between orientational domains of the RW-AFM state; note that due to a different sample bias voltage the domain walls appear dark in this measurement. (Measurement parameters: $U = +500\,\mathrm{mV}$, $I = 1\,\mathrm{nA}$; $T = 10\,\mathrm{K}$). **b**, Constant-current STM image of Ar bubbles in the uncovered Ir(111) surface ($\Delta z = 1.2$ nm). After the final Ar-ion etching step the Ir crystal was annealed at a reduced temperature of $T \approx 1300\,\mathrm{K}$ twice for 40 seconds each, i.e., the same procedure as for the sample preparations shown in the main text figures, only without subsequent deposition of Mn. (Measurement parameters: $U = +250\,\mathrm{mV}$, $I = 0.5\,\mathrm{nA}$; $T = 300$,K). **c**, Constant-current STM image of the Mn double-layer on an Ir(111) surface with Ar bubbles ($\Delta z = 144$ pm); bright lines indicate domain walls between orientational domains of the RW-AFM state, which form a network induced by the strain around the Ar bubbles. (Measurement parameters: $U = +10\,\mathrm{mV}$, $I = 1\,\mathrm{nA}$; $T = 4\,\mathrm{K}$). We would like to note that we always see the Ar bubbles as protrusions, regardless of the bias voltage. This is true not only for the Ar bubbles below the pristine Ir(111) surface, but also when the Ir surface is covered by a Mn double- or triple layer. Therefore we conclude that the measured apparent height reflects the topography and that the Ar bubbles induce a 'hill-like' structure similar to that proposed by Gsell et al. [4], see sketches in Fig. S7a,b.

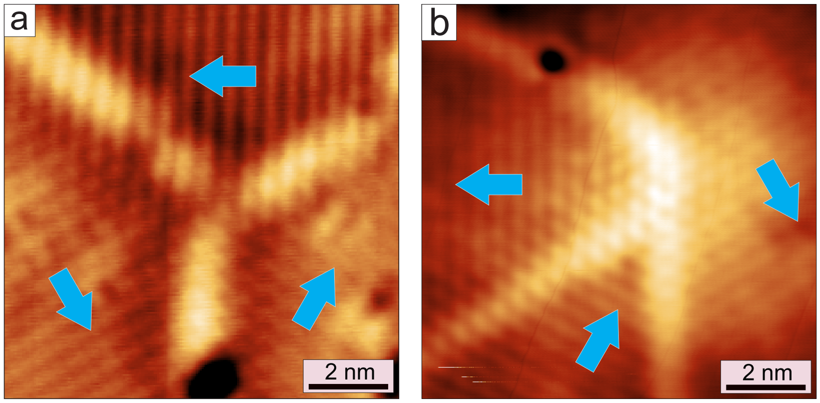

Figure S2. **Additional data demonstrating the occurrence of the 3Q state at the triple-junctions.** **a**,**b**, Constant-current SP-STM of a Y- and a T*-junction, respectively (a: $\Delta z = 21\,\mathrm{pm}$, b: $\Delta z = 44\,\mathrm{pm}$), exhibiting the hexagonal magnetic pattern of the 3Q state in their centers. The cyan arrows indicate the structural shift direction for the different orientational domains. (Measurement parameters: a: $U = +10\,\mathrm{mV}$, $I = 1\,\mathrm{nA}$, $B = 0\,\mathrm{T}$; b: $U = +10\,\mathrm{mV}$, $I = 7\,\mathrm{nA}$, $B = +2.5\,\mathrm{T}$; both: Fe-coated W-tip, $T = 8\,\mathrm{K}$.)

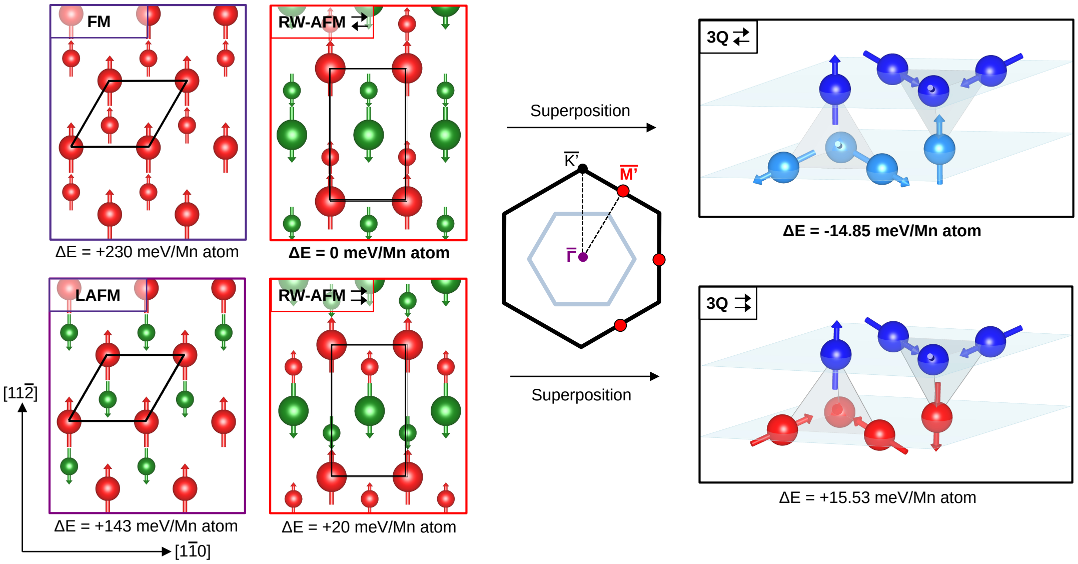

Figure S3. **Possible collinear and noncollinear spin configurations for hcp-Mn/hcp-Mn/Ir(111)** (see main text for details). For each collinear spin state, the energy difference after structural relaxation with respect to the energetically lowest RW-AFM$_{\rightleftarrows}$ state is given, while the energies of the two noncollinear 3Q superposition states (3Q$_{\rightleftarrows}$ and 3Q$_{\rightrightarrows}$) are given in relation to their corresponding single-Q building block. The high symmetry points of the two-dimensional hexagonal BZ which correspond to the single-Q FM and RW-AFM$_{\rightleftarrows}$ (RW-AFM$_{\rightrightarrows}$) state are marked in the respective color. Large (small) spheres in the illustrations of the collinear spin textures denote Mn atoms of the surface (subsurface) layer. For each collinear state the magnetic unit cell is indicated by a thin black line. Note that their magnetic moments are drawn in-plane for the sake of clarity, while they are pointing out-of-plane in the actual DFT calculation (the energetic preference for the in-plane alignment of the Mn spins only occurs upon the inclusion of SOC, i.e. by the MAE).

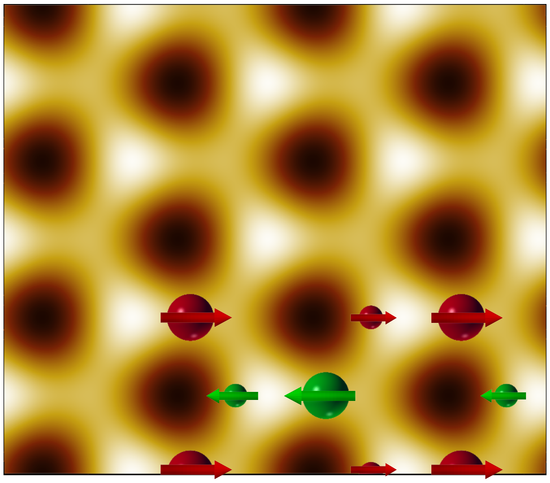

Figure S4. **STM simulation for the unshifted Mn double layer on Ir(111).** STM image calculated via DFT for the unshifted Mn double layer on Ir(111) in the RW-AFM$\rightleftarrows$ state (cf. Fig. 3c of the main paper). Large (small) spheres indicate atoms of the top (bottom) Mn layer. The STM image was calculated for an energy window corresponding to a bias voltage of +100 meV and at a height of 3 Å above the surface.

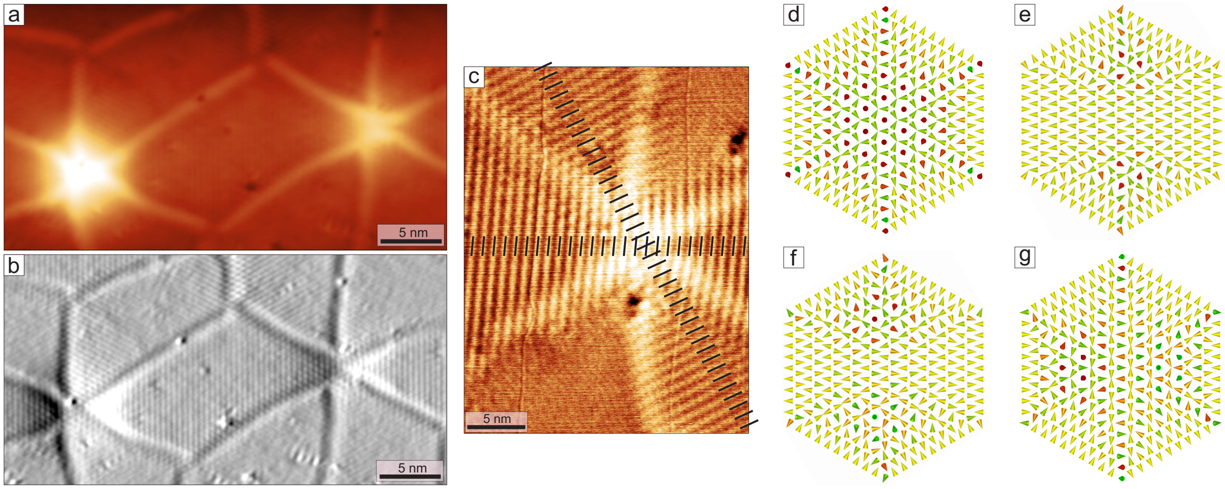

Figure S5. **Hexa-junction spin texture at the Ar bubbles.** **a**, SP-STM constant-current image of two hexa-junctions in the domain wall network (same data as displayed in Fig. 6a, slightly larger field of view, $\Delta z = 150\,\mathrm{pm}$). **b**, Current map obtained simultaneously to the SP-STM image of (a), in which the hexagonal magnetic pattern in the center of the hexa-junctions can be seen ($\Delta I = \pm 150\,\mathrm{pA}$). (Measurement parameters: $U = +10\,\mathrm{mV}$, $I = 1\,\mathrm{nA}$, $T = 4\,\mathrm{K}$, Cr-bulk tip). **c**, SP-STM constant-current image of a different hexa-junction, after a highpass filter with a cut-off frequency corresponding to 0.55 nm was applied to the raw data in order to remove the height variation of the Ar bubble and enhance the visibility of the magnetic state (now $\Delta z = 4$ pm). Equally spaced black lines indicate the maxima of magnetic contrast of the RW-AFM and demonstrate that two pairs of RW-AFM domains across the Ar bubble are in-phase; no magnetic contrast was obtained for the third orientational domain. (Measurement parameters: $U = +10\,\mathrm{mV}$, $I = 1\,\mathrm{nA}$, $T = 8\,\mathrm{K}$, Fe-coated W tip.) **d**, Spin model with six RW-AFM domains, with the constraint that three pairs of RW-AFM domains across the center are in phase, resulting in a perfect 3Q state in the center of the hexa-junction; the hexagonal magnetic pattern of the hexa-junction in (c) and the spatial distribution thereof are in good agreement with this spin model. **e**, Spin model with six RW-AFM domains, with the constraint that again three pairs of RW-AFM domains are in phase, resulting in a different spin texture in the center of the hexa-junction, which can be better described as a pair of T-/T*-triple-junctions; a T-/T*-triple-junctions is present at the right side of the measurement in (a), but it has not been possible to identify the spin texture at its center. **f**,**g**, Spin models with six RW-AFM domains, with the constraint that some pairs of RW-AFM domains are out of phase, resulting in pairs of T-/T*-triple-junctions with complex spin textures in their centers.

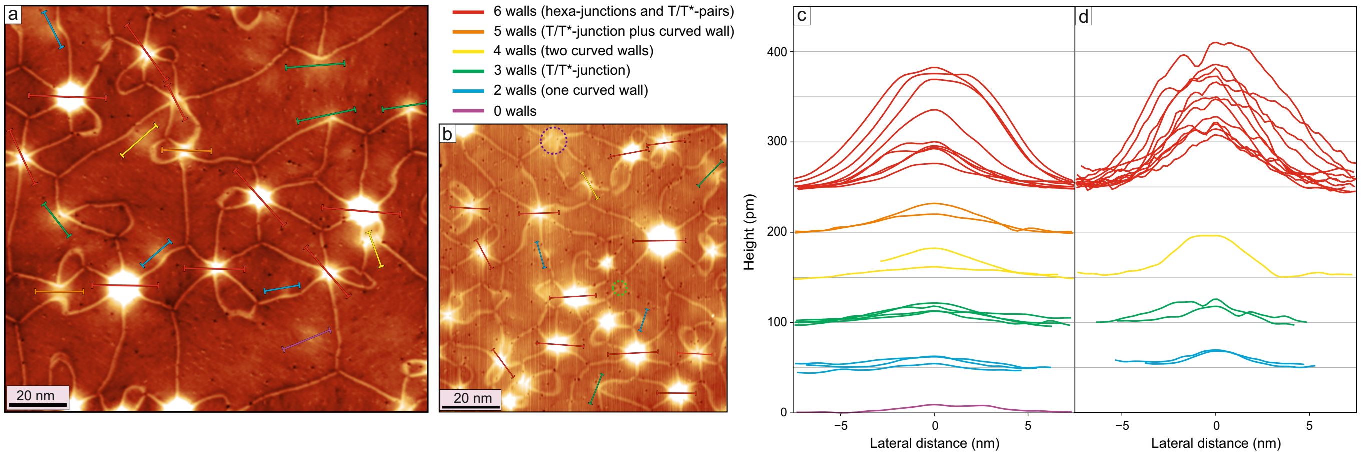

Figure S6. **Statistics on Ar bubble size and number of emerging domain walls.** **a**,**b**, STM constant-current images of two different sample preparations of the Ar bubble induced antiferromagnetic domain wall network in the Mn double-layer on Ir(111) (Fig. 1a and Fig. S1c, respectively). The colored lines indicate the positions of height profiles across Argon bubbles. The color code is based on the number of domain walls emerging from the respective Ar bubble, as detailed in the figure. The dashed green circle indicates a T-junction without an Ar bubble; the dashed purple circle indicates a comparably small Ar bubble with a pair of T-/T*-triple-junctions that are very far apart. **c**,**d**, Corresponding height profiles taken across the Argon bubbles according to the color code used in a,b. The plots are shifted vertically for clarity according to the number of walls emerging from the Ar bubbles. With an increasing size of the Ar bubbles more emerging domain walls are found.

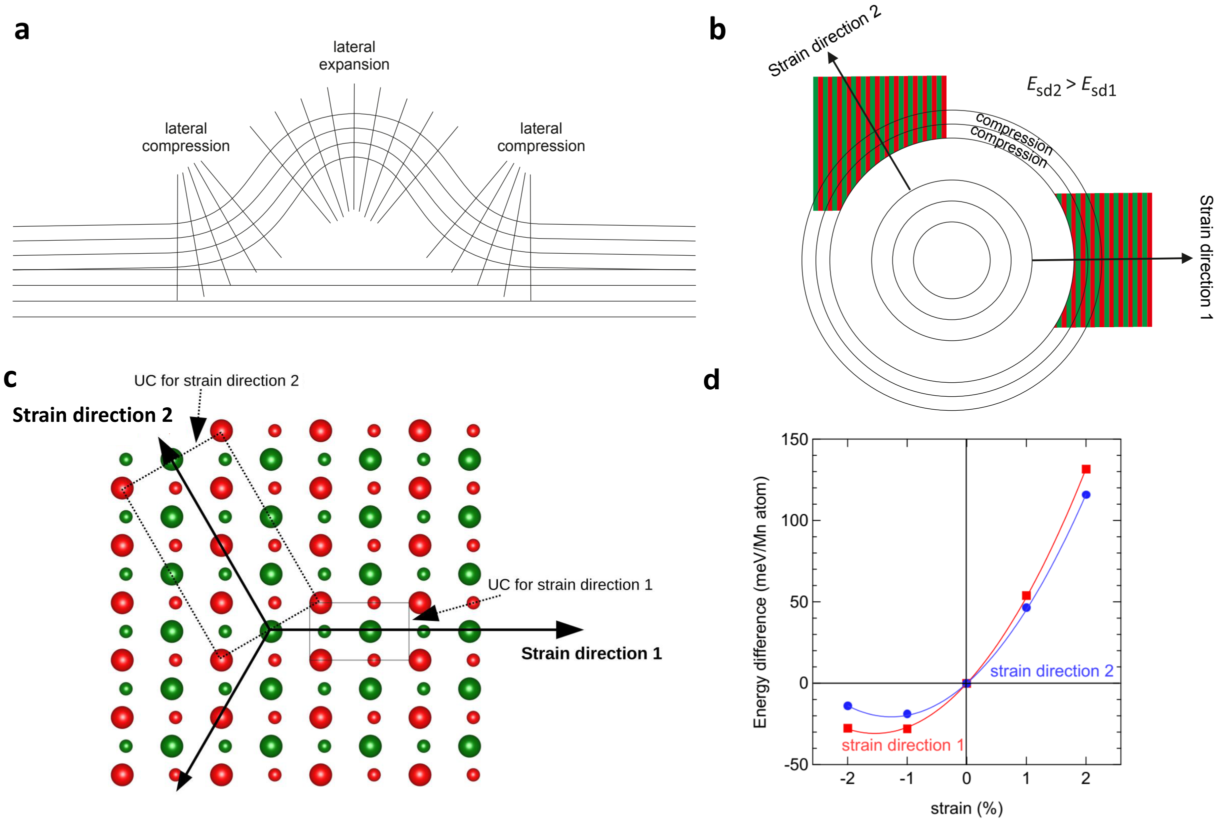

Figure S7. **Impact of strain on the row-wise antiferromagnetic state in the Mn DL on Ir(111).** **a**, Side view sketch of the local strain created in the film due to an Ar bubble in the substrate. This panel is inspired by the work of Gsell *et al.* [4]. **b**, Top view sketch of an Ar bubble together with two different configurations of an adjacent RW-AFM domain: the right domain has the rows of parallel spins tangential to the Ar bubble, whereas the top left one does not. This leads to two different strain directions (1 and 2) with respect to the RW-AFM state. **c**, Sketch of the geometry used for the DFT calculations including lateral strain (see also computational details). The laterally relaxed structure of the Mn double layer on Ir(111) is displayed. Red (green) spheres denote Mn atoms with magnetic moments pointing upwards (downwards). Large spheres denote top Mn layer atoms while small spheres indicate the Mn atoms of the bottom layer. The two considered strain directions are indicated by arrows and the two-dimensional unit cells (UC) used for the calculation are given. **d**, Total energies calculated via DFT for positive and negative strain values are displayed with respect to the unstrained case for both strain directions. The lines are obtained by a parabolic fit of the total energies. Note that there is an energy gain for compressive strain which is larger for strain direction 1 due to the exchange interaction which prefers antiferromagnetic alignment of Mn moments within the top and bottom layer. Upon applying compressive strain in direction 1 the distance to four nearest neighbor Mn atoms (in both top and bottom layer) with favorable antiparallel magnetic moments decreases which leads to a decrease in exchange energy. For strain direction 2, only two of the four nearest-neighbor Mn moments are antiparallel while two are parallel, i.e. aligned in the unfavorable direction. Therefore, compressive strain direction 2 is energetically less favorable.

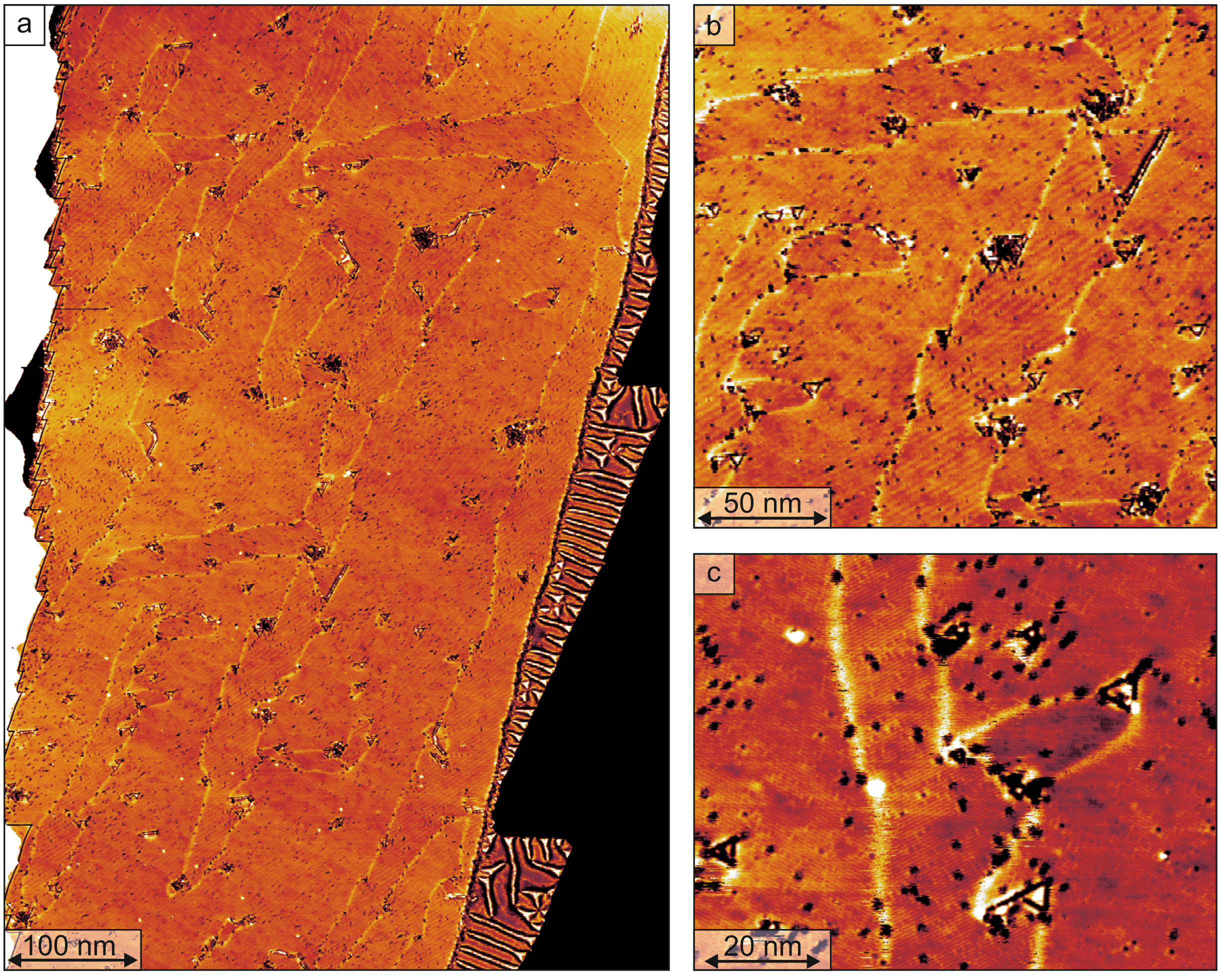

Figure S8. **Domain wall network induced by defect lines, measured at $T = 80\,\mathrm{K}$. a**, Constant-current STM image of an extended Mn double-layer area (center), with some areas of Mn monolayer and Mn triple-layer on neighboring terraces. This sample is free from Ar-bubbles, but the growth temperature of the Mn has been chosen to induce the formation of local defect lines. **b**, Enlarged view of a double-layer area of a. **c**, Closer view of defect line triangles at a different sample position (note that this image is rotated by 22° with respect to a,b). While these local defects also generate domain walls, imaged as bright lines at this bias voltage, the domain wall network neither shows very straight domain walls nor the high connectivity as it is observed in the Ar-bubble induced domain wall networks. (Measurement parameters: $U = -50\,\mathrm{mV}$, $I = 1\,\mathrm{nA}$, $T = 80\,\mathrm{K}$, Cr-bulk tip).

Table S1. DFT calculated total energies of the structurally optimized ferromagnetic (FM) state, layered antiferromagnetic (LAFM) state and the two row-wise antiferromagnetic states (RW-AFM$_{\rightleftarrows}$ and RW-AFM$_{\rightrightarrows}$) with respect to the energetically lowest RW-AFM$_{\rightleftarrows}$ reference state for Mn double-layers on Ir(111). Structural optimizations have been carried out using the `VASP` code and a symmetric slab comprising 9 Ir substrate layers and a Mn DL on each side of the film. All values are given in meV/Mn atom.

System	FM	LAFM	RW-AFM$_{\rightleftarrows}$	RW-AFM$_{\rightrightarrows}$
fcc-Mn/hcp-Mn/Ir(111)	201	167	0	21
hcp-Mn/hcp-Mn/Ir(111)	232	146	0	23

Table S2. Magnetic moments of the four considered collinear spin configurations for the two Mn double-layers on Ir(111) after structural relaxation. All values are given in units of μ_B.

System	magnetic layer	FM	LAFM	RW-AFM$_{\rightleftarrows}$	RW-AFM$_{\rightrightarrows}$
fcc-Mn/hcp-Mn/Ir(111)	top Mn	3.53	3.20	3.51	3.35
	bottom Mn	2.47	2.61	2.72	2.46
hcp-Mn/hcp-Mn/Ir(111)	top Mn	3.54	3.04	3.45	3.32
	bottom Mn	2.39	1.97	2.57	2.27

Table S3. Relaxed structural parameters for Mn double-layers on Ir(111) in the RW-AFM$_{\rightleftarrows}$ state. Structural optimizations have been carried out using the `VASP` code and a symmetric slab comprising 9 Ir substrate layers and a Mn DL on each side of the film. The relaxation of the shifted hcp-Mn/hcp-Mn/Ir(111) system has been done applying an asymmetric slab with 9 Ir layers and an enlarged 24-atomic rectangular magnetic unit cell. All values are given in Å.

System	$a_{\mathrm{in,Ir}}$	d_{Mn1Mn2}	d_{Mn2Ir1}	d_{Ir1Ir2}	d_{Ir2Ir3}	$d_{\mathrm{Ir,bulk}}$
fcc-Mn/hcp-Mn/Ir(111)	2.75	2.092	2.053	2.259	2.220	2.25
hcp-Mn/hcp-Mn/Ir(111)	2.75	2.060	2.043	2.265	2.222	2.25
System	d_{Mn1Mn2}	d_{Mn2Ir1}	d_{Ir1Ir2}	d_{Ir2Ir3}	$d_{\mathrm{lat.,topMn}}$	$d_{\mathrm{lat.,bottomMn}}$
hcp-Mn/hcp-Mn/Ir(111)$_{\mathrm{shifted}}$	1.970	2.047	2.264	2.221	1.056	0.085

Table S4. Lateral relaxed structural parameters for Mn double-layers on Ir(111) in different collinear magnetic states. Structural optimizations have been carried out using the `VASP` code, an asymmetric slab comprising 9 Ir substrate layers, a Mn DL on top and an enlarged 24-atomic rectangular magnetic unit cell. Values for the lateral shifted distances are given in Å, energy differences ΔE with respect to the corresponding symmetric state in meV/Mn atom.

System	state	$d_{\mathrm{lat.,topMn}}$	$d_{\mathrm{lat.,bottomMn}}$	ΔE
hcp-Mn/hcp-Mn/Ir(111)	FM	0.00	0.00	0.00
	LAFM	0.00	0.00	0.00
	RW-AFM$_{\rightleftarrows}$	1.06	0.09	−57.36
	RW-AFM$_{\rightrightarrows}$	0.26	0.06	−17.13

[1] A. Rodríguez-Sota, V. Saxena, J. Spethmann, R. Wiesendanger, R. Lo Conte, A. Kubetzka, and K. von Bergmann, Phase coexistence of Mn trimer clusters and antiferromagnetic Mn islands on Ir(111), ACS Nano **18**, 3699 (2024).

[2] J. Spethmann, S. Meyer, K. von Bergmann, R. Wiesendanger, S. Heinze, and A. Kubetzka, Discovery of magnetic single- and triple-Q states in Mn/Re(0001), Phys. Rev. Lett. **124**, 227203 (2020).

[3] M. Gutzeit, *Nanoscale spin structures at surfaces driven by exchange frustration and higher-order interactions*, Ph.D. thesis, Christian-Albrechts-Universität zu Kiel, Germany (2023).

[4] M. Gsell, P. Jakob, and D. Menzel, Effect of substrate strain on adsorption, Science **280**, 717 (1998).
